# Concomitant CNVs in healthy carriers with 7q31.1 microdeletions may suppress intellectual disability and autism spectrum disorders phenotype

**DOI:** 10.1192/j.eurpsy.2022.968

**Published:** 2022-09-01

**Authors:** E. Belyaeva, A. Kashevarova, G. Drozdov, D. Fedotov, I. Lebedev

**Affiliations:** Research Institute of Medical Genetics, Tomsk National Research Medical Center, Russian Academy of Sciences, Laboratory Of Ontogenetics, Tomsk, Russian Federation

**Keywords:** intellectual disability, IMMP2L, CNV

## Abstract

**Introduction:**

About 66% of chromosomal microdeletions and microduplications associated with pathological conditions are inherited [Smajlagić D. et al., 2021]. The mechanisms of incomplete penetrance and variable expressivity of CNV are not fully understood. The presence of concomitant CNVs in the genome of healthy parents may have a modifying effect.

**Objectives:**

Identification of additional CNVs in healthy carriers with 7q31.1 microdeletions.

**Methods:**

CNVs were revealed by Agilent Technologies 60K microarray and confirmed by qPCR.

**Results:**

We examined 3 families with inherited 7q31.1 microdeletions affecting only the *IMMP2L* gene, which is associated with intellectual disability, developmental delay and autism spectrum disorders. Family 1: Proband has intellectual disability, developmental delay, sensorimotor alalia. Microdeletion was inherited from the father, and a healthy sibling is also a carrier of rearrangement. In sibs, additional CNVs were identified: arr[hg19]: 4q31.21(144722583_144939143)×3; 9p12p11.2(43588066_43836428)×3; 16p11.2(32066967_33773163)×1; and 17q21.31(44199517_44577208)×3. Family 2: Proband suffers from development delay, speech disorder and autism. Microdeletion was of paternal origin. The father additionally demonstrated microduplication 16p11.2p11.1(33967926-35204414)×3. Family 3: Proband was diagnosed with development delay and cerebral palsy. The mother is a carrier of a similar 7q31.1 microdeletion; two concomitant CNVs were identified in her karyotype: 9p13.1(39176840_40614884)×3; and 16p11.2p11.1(32833891_35204414)×3. Thus, healthy parents in 3 families have CNV in a common region 16p11.2, which contains the *TP53TG3* gene. It is important that *TP53TG3* expression is associated with epistatic CNV-CNV interactions [Sun, Kardia 2010].

**Conclusions:**

Multiple CNVs in apparently healthy carriers of *IMMP2L* microdeltions may suppress disease phenotype due to the epistatic CNV-CNV interaction. This study was supported by Russian Science Foundation, grant no. 21-75-00112.

**Disclosure:**

No significant relationships.

